# “Fill the World with Love”: Songs with Prosocial Lyrics Enhance Online Charitable Donations among Chinese Adults

**DOI:** 10.3390/bs13090739

**Published:** 2023-09-04

**Authors:** Mei Hong, Dapeng Liang, Teng Lu

**Affiliations:** School of Management, Harbin Institute of Technology, 92 Xidazhi Street, Nangang District, Harbin 150001, China

**Keywords:** prosocial songs, general learning model, empathy, online charitable behavior

## Abstract

Research has shown that songs with prosocial lyrics can enhance individual prosociality. Building on the general learning model (GLM), this study demonstrated, through real-world charitable organizations, how songs with prosocial lyrics influence helping behavior that uses time as a currency. In Study 1, participants were randomly assigned to conditions of prosocial songs, prosocial lyrics, or neutral songs, and they were instructed to complete an online charity task. The results indicated that compared to the neutral songs, participants listening to prosocial songs and lyrics spent more time donating rice to the United Nations World Food Programme. This effect was replicated in Study 2, employing different media exposure from Study 1 (i.e., listening to background music). Furthermore, investigations into the underlying mechanisms revealed that this effect was mediated by interpersonal empathy. In summary, current findings suggest that songs with prosocial lyrics increase interpersonal empathy, subsequently influencing people’s online charitable donation behaviors in daily life.

## 1. Introduction

Prosocial songs and their influence on prosocial behavior is a captivating research direction within music psychology. Characteristic features of this kind of music are lyrics advocating kindness, sharing, compassion, and positive social interactions. It is believed to significantly impact prosocial behavior, i.e., behavior aimed at helping others. As proposed in the media effects model [1], exposure to music should be a stimulus for cognitive and behavioral consistency. Numerous studies have delved into the potential effects of prosocial songs on helping behavior. For instance, recent studies indicate that exposure to prosocial songs increases the accessibility of prosocial thoughts, promotes empathy, and enhances helping [2,3,4,5]. In a field experiment, Ruth (2017) [6] examined whether individuals exposed to songs with prosocial lyrics would purchase more fair-trade products, thus demonstrating greater prosocial behavior, compared to those who listened to songs with neutral lyrics. Further studies have also shown that listening to prosocial songs can reduce aggression and variables related to aggression [7,8]. Exposure to songs with prosocial content even reduced participants’ risky driving behavior [9] and reduced prejudice and discrimination [10,11]. Overall, prosocial songs have been proven to influence human behavior profoundly [12].

While increasing research suggests that songs with prosocial content positively affect people’s behavior, little is known about how this influence manifests or operates in real life. The significance of examining this issue lies in how research often applies theories of why individuals act altruistically to real-world charitable acts. For example, empathic altruism [13] and competitive altruism [14] have been utilized to explain individual donation behaviors in real-world charities [15] and marathon charity fundraising [16]. On the other hand, social information [17], peer observation [18], conformity [19], and public recognition [20] have been shown to positively influence individual charitable donations. This underscores the importance of applying these psychological theories to help understand and promote daily charitable activities.

However, a potential limitation of the existing research on prosocial songs is that past prosocial or altruistic research typically uses monetary donations as the currency of charitable behavior. While this is common, it is not the only means through which charitable behavior occurs, and this narrow focus may limit applications to other currencies. For example, studies have also utilized non-financial tasks such as blood donation [21], volunteer service [22], and mask wearing during pandemics [23] to empirically test prosocial behavior theories.

Moreover, these studies mainly focus on assumed or artificially created scenarios where helping behavior emerges. Therefore, it is necessary to apply research on prosocial songs to real-world scenarios, such as actual charitable causes where the need is not for currency. This is the aim of this study. This study will test the established theory of the prosocial songs–altruism hypothesis: prosocial songs will increase prosocial thoughts, feelings, and behaviors compared to neutral ones. To achieve this objective, this present study will investigate whether prosocial songs lead people to engage in more altruistic behavior, which has been shown to happen previously [2,3,4], through innovatively using real-world online charitable organizations. This will be compared to the level of altruistic behavior when exposed to neutral songs.

In this study, altruistic behavior will be measured through a unique real-world online charitable organization where the cost expended by donors is time rather than money. This is a particularly relevant behavioral change, as according to the general learning model (GLM, Buckley & Anderson, 2006 [24]), the explanation for prosocial songs leading to altruism is that songs with prosocial lyrics affect prosocial thoughts, empathy, and eventually prosocial behavior. Importantly, interpersonal empathy plays a mediating role in the influence of prosocial songs on prosocial behavior [2], and this positive emotion can alleviate negative emotions and feelings of altruists [25,26]. Unlike previous research, by examining the time spent on a charitable task, this study will be able to assess how prosocial songs promote prosocial behavior over a longer period, during which the relief of negative emotions may be slower because the goal of alleviating the suffering of those in need by altruists has not been reached [27]. Based on the GLM, individuals listening to prosocial songs might exhibit altruistic actions corresponding to the prosocial contents described in the lyrics. Therefore, Study 1 proposes the first hypothesis:

**Hypothesis** **1.** *Individuals listening to prosocial songs will spend more time on online charitable behavior than those listening to neutral songs.*

However, it is currently unclear whether the lyrics themselves or the lyrics accompanied by music play a more significant role in influencing prosocial behavior. Thus, Study 1 also compares the effect on donation outcomes of listening to prosocial lyrics alone versus songs with prosocial lyrics. Referring to previous research [5], Study 1 proposed a second hypothesis:

**Hypothesis** **2.** *Listening to songs with prosocial lyrics positively affects online charitable behavior more than prosocial lyrics alone.*

Furthermore, research by Greitemeyer (2009a,2009b) [2,3] demonstrated that in a German sample, songs with prosocial lyrics can influence helping behavior, which is mediated through interpersonal empathy. Thus, to replicate their research results in a Chinese sample, Study 2 proposes the third hypothesis:

**Hypothesis** **3.** *Interpersonal empathy mediates the beneficial impact of prosocial songs on charitable behavior.*

## 2. Study 1

Study 1 replicated Experiment 3, reported by Greitemeyer (2009b) [2], with some modifications. First, Greitemeyer measured requested monetary donations, whereas the current study assessed spontaneous, unsolicited, non-monetary donations. Second, this study employed different Chinese songs. Lastly, Study 1 investigated whether listening to songs with prosocial lyrics or lyrics alone influenced donation behaviors. It is anticipated that listening to prosocial songs would promote online charitable actions compared to neutral songs. In addition, prosocial songs would impact online charitable behavior more than prosocial lyrics alone.

### 2.1. Participants

A total of 203 college students (107 females) participated in the study. They were randomly assigned to either the prosocial songs group (N = 68), the neutral songs group (N = 65), or the prosocial lyrics group (N = 70). All participants underwent individual testing. To ensure no differences in prosocial tendencies across groups, we asked each participant to complete a measure of prosocial tendencies [28] before the experiment. The analysis of variance (ANOVA) showed no significant difference in total scores across groups (prosocial songs group, M=77.47, SD=9.42; neutral songs group, M=75.35, SD=9.28; prosocial lyrics group, M=78.17, SD=9.83; $F\left(2,200\right)=1.58$, p=0.208, $\eta_{p}^{2}=0.02$. All participants possessed either normal vision or vision corrected to normal, and all were right-handed. All participants provided electronic informed consent regarding the purpose of the research and experimental procedures.

### 2.2. Materials

#### 2.2.1. Music

Building upon the selection criteria of previous research [5,29], five prosocial and five neutral songs were selected as the potential options. Thirty-five students were invited to rate the prosocial, arousal, and love levels of the selected songs on a 7-point Likert scale, ranging from 1 point (not at all) to 7 points (very much). Based on the overall scores, two prosocial songs (“Devotion of Love [爱的奉献]” and “Fill the World with Love [让世界充满爱]”) and two neutral songs (“Cucurbit Flute [月光下的凤尾竹]” and “South of the Clouds [彩云之南]”) were selected. All four songs were in Chinese and had the same length after editing. Paired-sample t-tests revealed a significant difference in the prosocial level between songs with prosocial lyrics (M=5.77, SD=1.21) and songs with neutral lyrics (M=4.63, SD=1.29), $t\left(1,34\right)=-4.29$, p<0.001, Cohen’s d=−0.73, 95% CI =[−1.07,−0.38]. Conversely, there was no significant difference in arousal and love levels—arousal: $t\left(1,34\right)=-1.33$, p=0.193; love: $t\left(1,34\right)=-0.89$, p=0.378.

Prosocial songs. “Devotion of Love” is a classic song. The song’s lyrics adopt a progressive prose style to deepen the emotion of the theme of “love”. The song’s lyrics, “As long as everyone gives a little love, the world will become a better place”, has a unique and touching calling power.“Devotion of Love” is not only popular, but has also become the label of public welfare songs; when the country and the people need warmth and touching, the melody of this song will always ring out. “Fill the World with Love” is a Chinese charity song. Its lyrics like “Gazing deeply into your eyes, no need for more words, holding your hand tightly, this warmth remains unchanged” are filled with positivity and hope, conveying a simple yet powerful message: to fill the world with love. The lyrics emphasize the importance of education, learning, understanding, and passing on love. We can improve the world, overcome differences, understand others, and share peace and prosperity through love.

Neutral songs. The background for creating “Cucurbit Flute” was to promote the “Marriage Law” in the Dai ethnic region. The lyrics, “The golden peacock following the golden deer, heading together towards the green mist”, depict two lovers eventually getting married. “South of the Clouds” is a classic Chinese folk song that describes the beautiful natural scenery and diverse culture of Yunnan Province. The term “South of the Clouds” is a nickname for Yunnan. In Yunnan, colorful clouds often appear, hence the name “South of the Clouds”. This song is widespread in Mainland China and has a high reputation among overseas Chinese communities. For more information on the songs, please see the Supplementary Materials.

#### 2.2.2. Online Charitable Task

Freerice (www.freerice.com, accessed on 21 April 2023) is a non-profit website owned and supported by the United Nations World Food Programme, ingeniously embodying the principle of “time is money”. The website offers multiple-choice questions covering a wide range of topics. For each correctly answered question, Freerice donates 10 grains of rice to the United Nations World Food Programme. Users can participate in answering questions infinitely, with every correct answer resulting in a donation of 10 grains of rice to the World Food Programme. Therefore, the participants’ time spent answering questions is translated into concrete actions of delivering food to those in need. During the user’s participation, the total quantity of donated rice is updated in real time on the right side of the screen, making it clear to the user how their time investment has been translated into tangible aid. Also, there is a visual image of a bowl gradually filling up next to this number, intuitively demonstrating the impact of the user’s time investment on those in need. In this experiment, participants were required to find the correct definition out of four words (for example, the meaning of “水” was given options: “Water”, “Tree”, “Sun”, “Moon”).

### 2.3. Procedure

The experiment was conducted over a week from 8 a.m. to 6 p.m. Upon arriving at the lab, participants were randomly assigned to an independent compartment. The experimenter read aloud the instructions for the experiment before it began. A double-blind, randomized controlled design was used to evaluate the effects of prosocial songs, neutral songs, and prosocial lyrics on online charitable behavior. The participants were blinded to group assignments and research hypotheses. The experimenters were blinded to treatment allocations. Participants were given a pair of over-ear headphones for the experiment. They were unaware of the purpose of the study. All participants reported hearing the songs or lyrics clearly and normally.

The experiment began with a standardized instruction screen followed by an electronic informed consent form. Participants were instructed to listen to songs or lyrics. Those in the prosocial group listened to songs with prosocial lyrics, the neutral group listened to songs with neutral lyrics, and the prosocial lyrics group listened to lyrics read by the experimenters. Subsequently, participants removed their headphones and answered the Freerice task on the screen. Participants were told they could play the game as much as they wanted. Once the participants opened the compartment door, indicating they had finished the test, the experimenters recorded the amount of rice donated.

### 2.4. Data Analysis and Results

**Figure 1:**
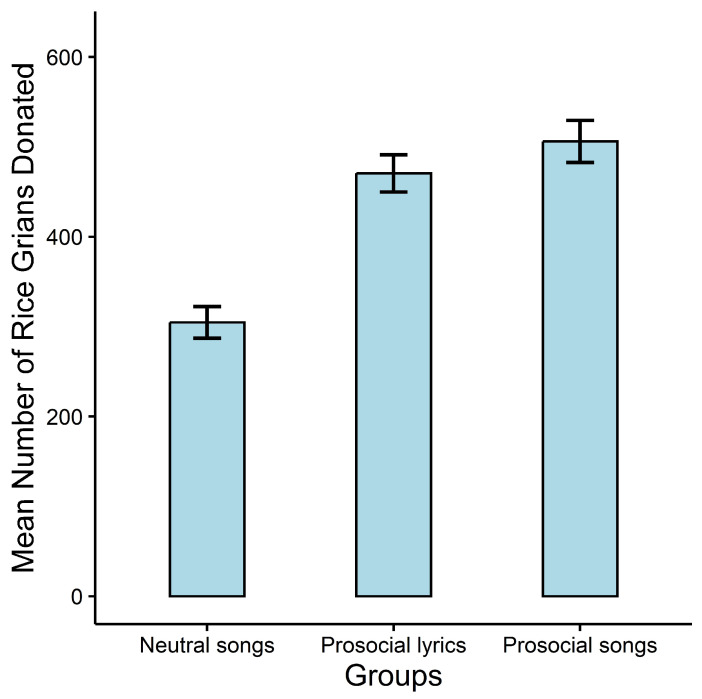
Mean number of grains donated across different groups in Study 1. Error bars represent one standard error of the mean (s.e.m.).

A two-way ANOVA was conducted with gender (female and male) and music type (prosocial songs, neutral songs, and prosocial lyrics) as the between-subjects variables, and the number of rice grains donated as the dependent variable. The results showed a significant main effect for music type, $F\left(2,197\right)=25.73$, p<0.001, $\eta_{p}^{2}=0.21$. Multiple comparisons (where we used Bonferroni adjustment) indicated that subjects in the prosocial group (M=506.03, SD=192.56) and the prosocial lyrics group (M=470.57, SD=173.46) donated significantly more rice than those in the neutral group (M=304.77, SD=141.48), both p<0.001. Meanwhile, the difference in in donating rice grains between the prosocial songs and the prosocial lyrics groups was not significant (p>0.05, see Figure 1). Neither the main effect of gender ($F\left(1,197\right)=0.03$, p=0.870, $\eta_{p}^{2}<0.01$) nor the interaction effect ($F\left(2,197\right)=0.11$, p=0.895, $\eta_{p}^{2}<0.01$) was significant.

## 3. Study 2

Study 2 was a replication of Study 1, with the following modifications. Firstly, Study 1 confirmed the powerful impact of attentively listening to prosocial songs on donation behavior. However, the effects of inattentively listening to songs have not been examined. Therefore, Study 2 used the same song list as Study 1 but played the songs in a background music manner. Secondly, Study 2 further explored whether the impact of music type on online charitable behavior was mediated through the affective pathway of the GLM. More specifically, the study hypothesized that interpersonal empathy mediated the beneficial effect of prosocial songs on donation behavior.

### 3.1. Participants

Drawing from the sample size utilized in prior prosocial song research [29,30], as well as the effect size and power (0.8) delineated in related studies [31], the sample size of this study was computed using G-Power 3.1.7, which established a target sample size of 64 participants per group. The study started with 142 recruited participants, and after the review process, 5 participants were excluded due to personal reasons. Finally, a total of 137 participants were recruited (72 females). Of these, 68 were randomly assigned to the prosocial group and 69 to the neutral group. All participants provided electronic informed consent for the experimental procedures.

### 3.2. Procedure

Study 2 used the same playlist and the Freerice task as Study 1. Participants were randomly assigned to either a prosocial or neutral songs group. To avoid interference from background music, experiments for both groups were conducted in quiet labs situated on separate floors. The experiment ran from 9 a.m. to 5 p.m. over two weeks. Based on the group allocation, prosocial or neutral songs were continuously broadcasted in the waiting area and the laboratory at a constant volume during the experiment. Participants filled out an Interpersonal Reactivity Index-C questionnaire. They then completed the Freerice task on the computer, and the experimenters recorded the final result.

The ability to empathize was measured by the Interpersonal Reactivity Index-C (IRI-C, Zhang et al., 2010 [32]). This questionnaire consists of 22 items, and participants were asked to rate each item’s description of themselves on a 5-point scale (1 = does do not describe me well; 5 = describes me very well). The average score formed an index, with higher scores on the Perspective Taking (IRI-PT), Empathic Concern (IRI-EC), and Personal Distress (IRI-PD) scales reflecting stronger empathy. The Fantasy (IRI-FS) scale was not included in the data analysis because it was irrelevant [33]. Cronbach’s α for this sample was 0.81 (IRI-PT was 0.80, IRI-EC was 0.53, IRI-PD was 0.80).

### 3.3. Data Analysis and Results

Empathy. Listening to prosocial songs increased empathy compared to neutral songs, $t\left(1,135\right)=3.16$, p=0.002, Cohen’s d=0.54, 95% CI =[0.20,0.88]. Participants who listened to prosocial songs reported more empathy (M=62.44, SD=9.30) than those who listened to neutral songs (M=57.16, SD=10.24).

**Figure 2:**
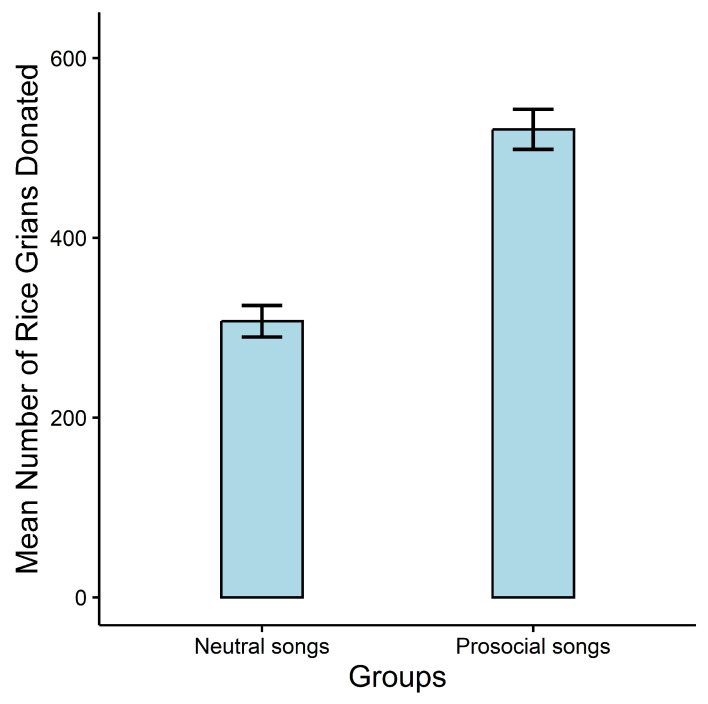
Mean number of grains donated across different groups in Study 2. Error bars represent one standard error of the mean (s.e.m.).

Online Donation Behavior. A two-way analysis of variance (ANOVA) was performed, with the number of donated grains serving as the dependent variable and gender (male and female) and type of song (prosocial and neutral songs) as the independent variables. Again, we identified a main effect of type of song ($F\left(1,133\right)=56.12$, p<0.001, $\eta_{p}^{2}=0.30$), but no main effect of gender ($F\left(1,133\right)=1.10$, p=0.295, $\eta_{p}^{2}<0.01$), or gender by music type interaction ($F\left(1,133\right)=0.68$, p=0.413, $\eta_{p}^{2}<0.01$), confirming that there were significant differences in donating rice between groups (prosocial song group, M=520.74, SD=183.84; neutral song group, M=307.25, SD=146.21, see Figure 2).

**Figure 3:**
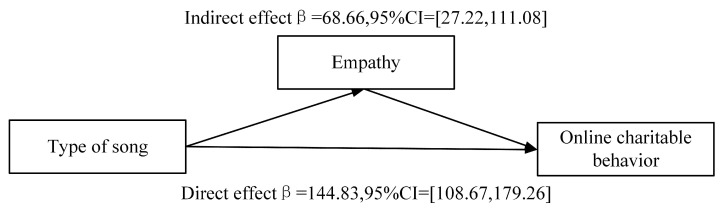
The mediation model demonstrates that the type of song indirectly affects online charitable behavior through empathy.

Mediation Analysis. To examine the mediating role of reported empathy, we conducted mediation analyses (PROCESS model 4, Hayes 2018 [34]) with music type as the independent variable, donated grains as the dependent variable, and reported empathy as the mediator. A bootstrap mediation analysis (5000 iterations) found that empathy mediated the relationship between type of song and prosocial behaviors (neutral song group = 0, prosocial song group = 1; bias corrected 95% confidence intervals excluding zero; see Figure 3. The results revealed that the indirect effects of music type on prosocial behavior through empathy were significant (β=68.66, SE=21.51, 95% CI =[27.22,111.08]). The direct effects of music type on prosocial behavior were also significant(β=144.83, SE=17.83, 95% CI =[108.67,179.26]).

## 4. Discussion

The results validate Hypothesis 1. Individuals exposed to prosocial songs spent more time donating rice than neutral songs. Thus, this finding supports previous research [2,3,4,6], i.e., prosocial songs can lead to altruistic behavior because it reveals that this influence exists in interactions with real-world charities, where the time spent is the donation requested. Furthermore, by using time as currency, this study demonstrates for the first time that prosocial songs can promote enduring prosocial behavior to mitigate negative emotions. These findings align with previous research on the empathy–altruism hypothesis [35] and the competition–altruism hypothesis [15].

However, the impact of prosocial songs did not lead to more charitable donations as predicted compared to listening to prosocial lyrics alone, thereby failing to support Hypothesis 2. Interestingly, participants exposed to prosocial lyrics dedicated more time donating rice to charity than those exposed to neutral songs. Why were prosocial lyrics successful in promoting prosocial behavior here as well? One reason might be that the experimenters read those prosocial lyrics aloud. The presence of others and the perceived social norms conveyed through the lyrics could have influenced participants’ responsiveness to prosocial messages [36,37]. While listening to the lyrics, social pressure or the desire to conform to societal expectations might have facilitated online charitable behavior. Another reason is that the prosocial songs selected in Study 1 were all familiar to Chinese listeners, possibly reducing the novelty and impact of the songs [38]. Conversely, presenting the lyrics differently in the absence of music might generate unique cognitive processes that draw attention to the content of the lyrics [39]. Furthermore, well-crafted prosocial lyrics can evoke empathy and a sense of justice even in the absence of music [40], and that subsequent prosocial behaviors can mitigate these emotions [2,3]. Therefore, it is not surprising that similar outcomes were observed for time spent donating rice to charity among those who listened to prosocial songs or lyrics, both of which exceeded the time spent by those who listened to neutral songs. These findings are inconsistent with a study by Yu et al. (2019) [5], who found that songs with prosocial lyrics had a more significant positive impact on college students’ willingness to participate in unpaid experiments than merely reading the lyrics. However, their methodology involved reading the prosocial lyrics rather than listening, and it was set in a hypothetical prosocial context. Whether these differences led to the disparate findings requires further investigation.

Hypothesis 3 received empirical support from Study 2, demonstrating that prosocial songs played in participants’ environments were positively associated with online charitable donations. This contrasts previous research where experiments generally employed hypothetical or contrived settings, requiring participants to focus explicitly on the songs. In Study 2, the charitable acts were authentic, and the songs served as background while participants engaged in the task. The findings suggest that even inattentively listening to prosocial songs could similarly activate prosocial thoughts and empathy [6,41], encouraging participants to devote more time to the Freerice task. Significantly, the prosocial songs–altruism hypothesis and potential mediating factors were examined across varying cultural backgrounds. This leaves the question of whether cognitive or emotional pathways truly mediate the relationship between media exposure and charitable actions in a Chinese sample. Study 2 supported and extended the research of Greitemeyer (2009a, 2009b) [2,3], indicating that in a Chinese cultural context, prosocial songs enhanced online charitable actions via an empathy-driven pathway, compared to neutral songs. Songs featuring prosocial lyrics can capture empathetic attention and inspire prosocial thoughts in listeners, ultimately leading to a favorable evaluation of donation behaviors. Consequently, participants exposed to the prosocial songs spent significantly more time donating grains to charitable organizations.

In terms of gender differences, previous research has found that females are more prosocial than males [42], including during preschool and adolescence [43,44]. Nevertheless, the current study indicates that adult female participants did not score higher than adult males in prosocial tasks, and there was no significant difference between the two. These results could be influenced by a small sample size or other factors, such as task type and developmental process. Eisenberg et al. (2005) [45] proposed that gender differences in prosocial tendencies may be related to the nature of the task at hand. In their meta-analysis examining gender and age differences among adolescents, females and males did not differ in instrumental helping behaviors (such as giving directions to others or giving up seats to strangers) or sharing and donating behaviors. In contrast, females score higher on moral reasoning related to caring [46], and gender differences favor females on prosocial behaviors as well as on some measures of empathy/compassion in childhood and adolescence [45]. Importantly, from a developmental perspective, a recent prosocial music study [47] and four cross-cultural experimental data set studies [48,49,50,51] suggest that gender differences in prosocial tendencies become progressively smaller as puberty ends. Thus, the lack of sex differences found in the current results reflects these issues.

## 5. Limitations and Future Directions

This study acknowledges several limitations that could potentially impact the interpretation of its findings. First, the experiment did not incorporate a neutral lyrics condition. Given the well-documented effects of prosocial lyrics as opposed to neutral lyrics [3,6,41], and with the primary dependent variable being prosocial donations, it was more efficient to focus solely on the two main factors: prosocial songs and lyrics. Second, the study’s results could be biased by participants’ preferences for the songs used. Liking and familiarity are interrelated factors in music [52]. Preliminary tests indicated varied preferences for the songs among participants. Future research might emphasize selecting familiar and unfamiliar songs but with no differences in liking or attempt to measure liking as a covariate. Third, the Freerice task is a knowledge test, which might elucidate why some individuals ceased playing, perceiving the game as too challenging rather than losing their motivation for prosocial actions. Nevertheless, given that the sample comprised college students, their vocabulary should be sufficient to meet the task’s demands, predicting only a marginal effect. Lastly, the current study is limited to short-term impacts; the effects of listening to prosocial songs were assessed in immediate cognitive, affective, and behavioral terms. According to the GLM, recurrent exposure to prosocial media could lead to long-term personality changes by developing and constructing knowledge structures [3,53]. Therefore, future research may benefit from employing a longitudinal paradigm to examine the sustained effects of prosocial media on social behaviors. Demonstrating these effects within dynamic social interactions would present a more compelling and meaningful accomplishment.

## 6. Conclusions

This research presents a form of charitable act that is economical and low-cost, an aspect likely prioritized by the general public. The findings complement prior studies on prosocial songs, showcasing a noticeable positive correlation between prosocial songs and charitable acts in the real world. These investigations delve into why prosocial songs can evoke and amplify prosocial behaviors across varied cultural backgrounds. In a Chinese cultural context, our research confirms that the prosocial songs–altruism hypothesis influences actual charitable donations, particularly when time, rather than money, is the requested currency. Hence, this current study offers significant implications for prosocial education in today’s society: listening to songs with prosocial lyrics heightens Chinese adults’ attention to prosocial-related information, thereby manifesting higher levels of altruism when confronted with situations demanding assistance from others. Notably, scholarly discussions on the influence of Chinese music on individuals are relatively scarce. By incorporating Chinese songs, this study examined the impact of music on people from an altruistic perspective, further enriching the genre of music studied. Additionally, future research could adopt similar methodologies to investigate other prosocial theories, such as effective altruism [54], reciprocal altruism [55], or kin selection theory [56]. Such investigations would elucidate why individuals spend time on charitable donations across different social contexts, thereby reaffirming the positive impact of these theories on modern society’s charitable endeavors.

## Data Availability

The data shown in this research are available on request from the corresponding author.

## Supplementary Materials

The following supporting information can be downloaded at: https://www.mdpi.com/article/10.3390/bs13090739/s1.
