# Supplementary material for: “Fill the World with Love”: Songs with Prosocial Lyrics Enhance Online Charitable Donations among Chinese Adults
Source: Behav Sci (Basel). 2023 Sep 4;13(9):739. doi: 10.3390/bs13090739 (PMC10525948; doi:10.3390/bs13090739)
Supplement: Supplementary file 1 [file behavsci-13-00739-s001.zip › behavsci-2546776-supplementary.pdf]

"Fill the World with Love": Songs with Prosocial Lyrics Enhance Online Charitable Donations among Chinese Adults

Supplementary Materials

These Supplementary Materials are associated with the paper *"Fill the World with Love": Songs with Prosocial Lyrics Enhance Online Charitable Donations among Chinese Adults* published in *Behavioral Sciences*.

1. Dedication of Love (Prosocial 1)

This is the call of the heart

This is love's devotion

This is the spring breeze on earth

This is the source of life

No more deserts of the heart

No more wilderness of love

Even death is deterred

The flower of happiness blooms everywhere.

As long as everyone gives a little love

the world will become a better place

2. Fill the World with Love (Prosocial 2)

Holding your face gently

Wipe away your tears for you

This heart will always be yours

Tell me I'm not alone anymore

Gazing deeply into your eyes

no need for more words

Holding your hand tightly

This warmth remains unchanged

We share the joy

We endure the same

We share the same expectations

We share the storm

We share the same quest

We share the same love

Whether we've known each other before

Whether in front of us or in the sky

I wish for you with all my heart

I wish you happiness and peace

3. Cucurbit Flute (Neutral 1)

Moonlight ah below the phoenix-tailed bamboo yo.

Gentle ah beautiful like a green mist yo;

The good girl in the bamboo building.

The girl in the bamboo building is as bright and colorful as a night pearl.

How many affectionate gourd sheng.

To you pouring out the heart of love and adoration.

Why don't you open your window?

The phoenix-tailed bamboo under the moonlight.

Soft and beautiful like a green mist;

The good girl in the bamboo building.

Her singing voice is as sweet as fruit dew.

The young man who is in love with you.

Don't get entangled in the betel nut tree;

My heart belongs to a man.

A golden peacock for a golden deer.

The moonlit bamboos

Soft and beautiful like a green mist;

The good girl in the bamboo house.

For whom she opens her door and window.

Oh, it's the little Yanpeng from the agricultural station.

Picking away the pearl of the night;

The golden peacock following the golden deer,

Heading together towards the green mist

4. South of the Clouds (Neutral 2)

South of the colored clouds, the direction of my heart

Peacocks fly away, memories are long

Jade Dragon Snow Mountain is shining with silver light

Lijiang, a beautiful color, I'm on the road

South of Colorful Clouds The place where I go back to

The fragrance of the past floats with the wind

Butterfly Spring, the song is flowing

Lugu Lake, my heart is still rippling.

I remember how blue the sky was.

Your eyes shone with gentle sunshine

The world is changing.

Where are you now?

Forgive me for not being able to walk with you for so long.

Other people's paradise is not our faraway place

Don't regret this trip

South of the colored clouds, the direction of my heart
